# Electron microscopy dataset for the recognition of nanoscale ordering effects and location of nanoparticles

**DOI:** 10.1038/s41597-020-0439-1

**Published:** 2020-03-25

**Authors:** Daniil A. Boiko, Evgeniy O. Pentsak, Vera A. Cherepanova, Valentine P. Ananikov

**Affiliations:** 1 Zelinsky Institute of Organic Chemistry, Russian Academy of Sciences, Leninsky Prospekt 47, Moscow, 119991 Russia; 2 Lomonosov Moscow State University, Chemistry Department, Leninskie Gory 1/3, Moscow, 119991 Russia

**Keywords:** Materials science, Nanoparticles, Scanning electron microscopy

## Abstract

A unique ordering effect has been observed in functional catalytic nanoscale materials. Instead of randomly arranged binding to the catalyst surface, metal nanoparticles show spatially ordered behavior resulting in formation of geometrical patterns. Understanding of such nanoscale materials and analysis of corresponding microscopy images will never be comprehensive without appropriate reference datasets. Here we describe the first dataset of electron microscopy images comprising individual nanoparticles which undergo ordering on a surface towards the formation of geometrical patterns. The dataset developed in this study spans three levels of nanoscale organization: (i) individual nanoparticles (1–5 nm) and arrays of nanoparticles (5–20 nm), (ii) ordering effects (20–200 nm) and (iii) complex patterns (from nm to μm scales). The described dataset for the first time provides a possibility for the development of machine learning algorithms to study the unique phenomena of nanoparticles ordering and hierarchical organization.

## Abstract (summary)

**Table:** 

Measurement(s)	Nanoscale Phenomena
Technology Type(s)	scanning electron microscopy
Factor Type(s)	ordered/unordered
Sample Characteristic - Environment	nanostructure

Machine-accessible metadata file describing the reported data: 10.6084/m9.figshare.11926929

## Background & Summary

Scanning electron microscopy (SEM) is widely used for materials characterization^1^. The microscopy images contain abundant information about the sample, because the brightness of a specific point depends on the spatial structure and physical properties^2^. Metal nanoparticles are particularly well detectable using electron microscopy^3^.

Nanoscale materials exhibit catalytic activity in the practically important cross-coupling^4^, oxidation^5^, electron transfer^6^, C-H activation^7^, and substitution^8^ reactions. Palladium nanoparticles deposited on the carbon surface are among the most demanded substances extensively studied by electron microscopy. As heterogeneous Pd/carbon catalysts, they are ubiquitously used in organic synthesis for production of drugs, pharmaceutical substances and molecular electronics devices^9–12^. Large scale production of Pd/C catalysts is utilized in industry. Understanding the structural arrangements of metal nanoparticles on the surface of catalytic materials is the key for controlling the activity and selectivity of catalysts, which is needed for optimization of industrial technologies^13^.

**Fig. 1:**
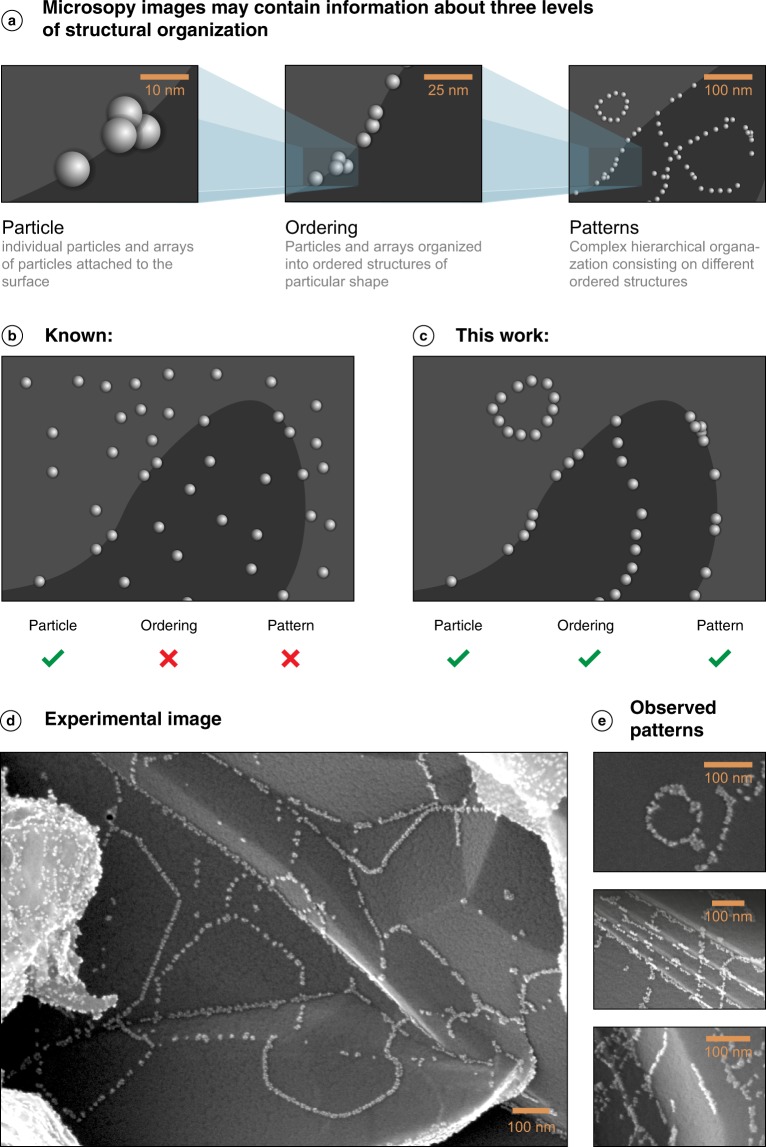
Different types of structural organization of nanoscale materials. (**a**) simulated images of components present in the dataset reported here, including individual nanoparticles, arrays of nanoparticles, ordered structures and patterns, (**b**) simulated image of the default random attachment and uniform distribution of nanoparticles at the surface; (**c**) simulated image of specific binding of nanoparticles with ordering effects and the appearance of patterns; (**d**) a real experimental microscopy image comprising the ordering of nanoparticles; (**e**) examples of real experimental images of observed patterns.

Three levels of structural organization from nano-scale to micro-scale can be accurately characterized by electron microscopy (Fig. 1a). Random binding of metal nanoparticles to the surface leads to more or less uniform spatial distributions and does not show ordering effects (Fig. 1b). In contrast, specific binding of particles gives ordered patterns (Fig. 1c). As an example, the illustrations in Fig. 1b,c comprise exactly the same numbers of particles but obviously different types of organization.

Caused by specific particle-surface interactions, ordering of metal nanoparticles and formation of specific geometrical arrangements/patterns has been observed in a variety of nanoscale materials^14–20^. However, it is difficult to isolate such unique structural arrangements in a stable form for collection of datasets. Recently, we have developed a special procedure to produce the ordered nanoscale materials in stable forms by using easily available reagents^21^. Optimized version of this procedure has made it possible to collect the dataset described in this study (see details in the Methods section below). The experimental microscopy images clearly show the ordering of metal nanoparticles and the formation of spectacular patters (Fig. 1d).

Here we present two datasets of SEM images of Pd nanoparticles deposited on a carbon surface:

(i) The first dataset contains 750 images of ordered nanoscale materials, where the attachment of nanoparticles in ordered way to the carbon surface shows some geometric patterns (see Fig. 1c as a model and Fig. 1d as an example). Different types of patterns can be observed in these images, e.g. lines, intersections, networks, ellipses and circles, among many other (Fig. 1e). Each recorded microscopy image may contain approximately 500–5000 nanoparticles. On the whole, the presented dataset contains information recorded for more than 1 million of nanoparticles.

The dataset reflects three levels of structural organization: individual nanoparticles and arrays of nanoparticles, ordered structures and patterns (Fig. 1a). The overall hierarchical scale for these objects ranges from 1 nm to ~100 μm. By spanning the five orders of magnitude size range, the dataset connects nanoscale and microscale phenomena and provides a possibility to visualize the ordering effects in materials.

(ii) The second dataset consists of 250 images of non-ordered nanoscale materials showing the random attachment of Pd nanoparticles (see Fig. 1b as a model). Drastic difference can be seen when comparing them with the images provided in the first dataset. Since this type of attachment (non-ordered) is well known, we provide a smaller dataset. This dataset is provided for comparative purposes (bearing in mind the development of machine learning projects in the future). The images in Datasets 1 and 2 were recorded with the same electron microscopy parameters to facilitate subsequent comparison and analysis.

It is important to note, that different carbon materials may exhibit different degrees of order in deposited particles’ positions. Moreover, due to the probabilistic character of the deposition process, particles may spontaneously show some deviations from the expected behavior (i.e. completely ordered samples may not be always expected, whereas predominantly ordered samples represent a better definition).

The mechanistic reasons for the formation of ordered structures deserve a brief note^21,22^. The surface of a carbon material may be considered as a superposition of graphene sheets and may contain regions of different chemical reactivity. The graphite sample utilized in this study contains a small amount of highly reactive regions, while the rest of the sample is less reactive and may be relatively inert.

Metal nanoparticles predominantly bind to the areas with high reactivity due to the larger binding energy^21^. Formation of such high-reactive regions on the carbon surface can be caused by specific character of surface formation or by defects — interruptions of the regular structure. Spatial positions of these sites follow certain regularities, which results in the effect of particle positions ordering. Ordered nanoparticles can form a variety of patterns indicating specific structural features associated with locations/boundaries/defects of individual graphene sheets. Thus, interacting with the material, the metal particles act as a contrasting agent, with the positions of the attached nanoparticles highlighting the areas of high chemical reactivity. Additional contrasting reveals structural features of the carbon material that would be difficult to distinguish without the attached metal nanoparticles^21^.

In terms of the charge/electron delocalization, the easiness of palladium nanoparticles attachment to the carbon material surface depends on the local electron density. Non-uniform electron density at the surface is reflected by the non-uniformity of palladium nanoparticles positioning. The regions of non-uniform electron density are called ‘defects’, for example, Stone-Wales defects (resulting from the presence of different crystallization centers during the formation of carbon material), sheet edges (that interact with palladium by dangling bonds or heteroatom groups), bends of carbon sheets, and point defects (the vacancies and adatoms in the material structure).

To distinguish between different types of defects, one may look at the underlying material. As a  typical case, the sheet edges form a small difference in height (a step) producing a contrast in the images recorded in secondary electron mode; sequences of steps usually form cascades of parallel lines. The differences in brightness are also useful for determination of the carbon sheet folds. Grain boundaries are identified by closed micro-sized contours. Single circles are probably formed by oxidation of point defects, which are also responsible for the presence of solitary nanoparticles^16,17^. Attachment of multiple particles at the same location leads to the formation of agglomerates.

## Methods

### Data collection

#### Electron microscopy

##### Principle of operation

The principle of operation of a scanning electron microscope is to scan the surface of a sample with the electron beam. Various types of signals are generated during the interaction of the electron beam with the sample substance: secondary electrons, backscattering electrons, and other signals detected by corresponding detectors^23^. To study the morphology of carbon materials, the most informative approach is to register images by the upper detector, where only secondary electrons are recorded.

Secondary electrons have low energy; their emission is possible only from the superficial layers of the sample, and their number depends on the angle of the collision of the electron beam with the surface of the sample. Therefore, registration of secondary electrons makes it possible to visualize the surface topography of the sample and to study its morphology. In addition, the secondary emission coefficient depends on the type of material. This causes differential contrasting of the particles of different nature, having different densities, conductivities, ionization energies, and consisting of elements with different atomic numbers^24^.

##### Sampling and equipment

Before the measurements, the samples were mounted on an aluminum specimen stub and fixed with a conductive graphite adhesive tape. The sample morphology was studied under native conditions to exclude the metal coating surface effects^25^. The observations were carried out using a Hitachi SU8000 field-emission scanning electron microscope (FE-SEM). The images were acquired in secondary electron mode at 10–30 kV accelerating voltage and a working distance of 6–12 mm. The EDX studies were carried out using an X-max EDX system (Oxford Instruments, UK).

##### Deposition of palladium on carbon surface

The model utilizes straightforward direct process of deposition of the metal on the carbon material, avoiding the sorption of impurities and additional reagents (for example, modifying chemicals). It is also important to avoid high temperatures, which can affect the sample morphology due to the interaction of metal particles with carbon^26^. For this reason, the Pd_2_dba_3_ complex is an appropriate choice as a precursor for the metal nanoparticles. It easily forms small nanoparticles in mild conditions under rapid heating to 50 °C. No more than 5 wt.% of the complex is required for deposition of nanoparticles onto a carbon material.

Three types of carbon materials were used in the experiments: (1) graphite powder extra pure grade (90% w/w of the particles smaller than 90 µm) for samples S1 and S2; (2) nanoglobular carbon (carbon black type T900, produced in the Institute of Hydrocarbon Processing, SB RAS, particle diameter 100–400 nm) for S3, and (3) pressed graphite bars 1.5 × 5 × 25 mm for samples S4, S5.

##### Preparation of the samples with ordered distribution

A screw cap tube was charged with Pd_2_dba_3_·CHCl_3_ (5 mg for both samples), graphite powder (100 mg) and 5 mL of CHCl_3_. The reaction mixture was stirred at 50 °C for 1 h. Subsequent filtration led to separation of the transparent solution and carbon material. For the measurements, the material was dried and washed with acetone to remove dba if necessary.

##### Preparation of the samples with disordered distribution

A screw cap tube was charged with Pd_2_dba_3_·CHCl_3_ (5 mg for S3, 0.4 mg for S4 and 8.5 mg for S5), nanoglobular carbon (for S3) or graphite bar (for S4 and S5) and CHCl_3_ (5 mL for S3; 4 mL for S4 and 8.5 mL for S5). The reaction mixture was stirred at 50 °C (for S3 and S4) or 70 °C (for S5) for 24 h. Before the measurements, the material was dried and washed with acetone to remove dba if necessary.

Sample S5 is a working catalyst; it has been used as a catalyst in the styrene hydrogenation reaction before being microscopically examined.

### Data verifications

#### NMR spectroscopy

NMR measurements were performed using a Bruker DRX500 spectrometer equipped with 5-mm BBO probe head operating at 500.1 and 125.8 MHz, a Bruker AVANCE 400 spectrometer operating at 400.1 and 100.1 MHz, or a Bruker Fourier HD300 spectrometer operating at 300.1 and 75.5 MHz for ^1^H and ^13^C, respectively, in CDCl_3_ or CD_2_Cl_2_. The spectra of reaction products were acquired immediately after the reactions and processed using TopSpin 3.5 software package. The ^1^H and ^13^C chemical shifts were referenced to internal standards provided by the solvent.

#### ICP-AES measurements

Palladium content was determined by ICP-AES (inductively coupled plasma atomic emission spectrometer) measurements using JY 38 (Jobin Yvon) spectrometer. The sample of Pd/C was melted with sodium persulfate, then transferred to a solution. Determination of palladium was carried out in the solution.

## Data Records

The two datasets consist of total 1000 images of the carbon materials with deposited palladium nanoparticles (Dataset 1 contains 750 images representing predominantly ordered nanoscale structures and Dataset 2 contains 250 images representing predominantly non-ordered nanoscale structures). The datasets are available at Figshare:

Dataset 1^27^: 10.6084/m9.figshare.11783661

Dataset 2^28^: 10.6084/m9.figshare.11783667

Each image is 1280 × 1024 pixels in size and presented in the TIFF format (.tif). Each image has a name to identify it with the provided data. The 134 px wide digital captions at the bottom indicate the accelerating voltage, the working distance, the magnification, the mode of operation, the type of detector, and the scale.

These indicators, along with other acquisition parameters and sample names, are available in a separate CSV file:

- Sample number
- Acceleration voltage, V
- Magnification
- Working distance, µm
- Emission current, nA
- Lens mode
- Area code

The study was carried out for different source materials. The materials are specified in the Table 1. Although, materials exhibit predominantly either ordering, or disordering behavior, images with some degree of order/disorder may be obtained for both types of the materials. We made our best effort to exclude any controversial image from the dataset as the presence of mixed-type images would make an analysis much more complicated.

**Table 1:** The ‘ordered’ and ‘non-ordered’ datasets are represented by images of varying magnification.

Dataset no.	Degree of ordering	Samples	Number of images	Magnification			Material
				50 k	100 k	200 k	
1	Predominantly ordered	S1, S2	750	31.6%	46.8%	21.6%	graphite powder extra pure grade
2	Predominantly disordered	S3, S4, S5	250	36.8%	39.6%	23.6%	nanoglobular carbon (S3), pressed graphite bars (S4, S5)

As future users may be interested in different levels of structural organization, we provide images with different magnifications. Some of them are images of the same area of a material’s surface. We marked these images with unique area codes in the “Area” column for each image. Lone images are marked as “no_area”. The images of the same area, but at different magnifications, have only partial overlap, this provides additional opportunities to train alignment/matching technics using this database.

**Fig. 2:**
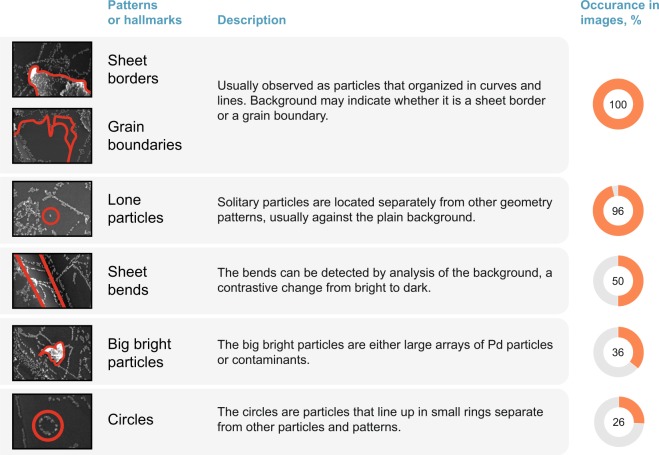
Types of patterns and structural features present in Dataset 1 (‘occurrence in images’ is the fraction of images containing the features of specified type). The percentage are provided for a random sample of size 50 from images with magnification 50 k and 100 k.

As described above, many different ordering patterns may be observed in the images of Dataset 1. A representative set of images was analyzed by visual inspection, and the presence of particular ordering patterns was deduced. The results of analysis of a random selection of 50 images are summarized in Fig. 2.

Overall, there are two types of features that are present in all or almost all of the images – sheet borders/grain boundaries and solitary particles. 50% of the images comprise bends of graphene sheets. Big bright particles are present in 36% of the images, while the circles are present in 26% of the images. The labels for the images are available in a separate CSV file.

It should be noted that Fig. 2 exemplifies only a fraction of geometrical patterns observed in this study. Many other types of ordered structures or geometrical arrangements may be also found in the images.

## Technical Validation

**Fig. 3:**
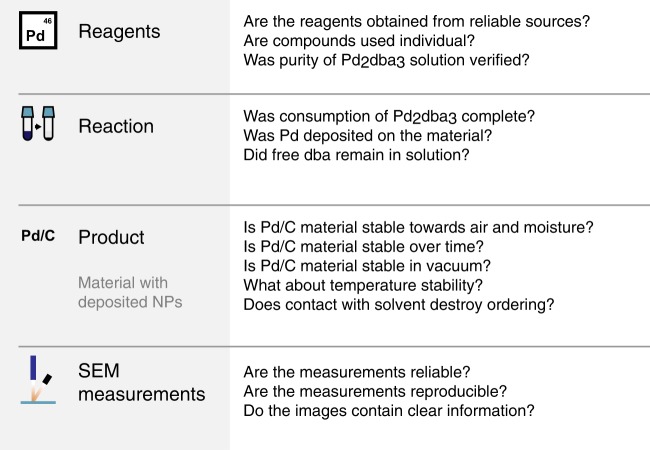
The validation framework. Key aspects were validated for reagents, reaction, product and measurements.

Thorough technical validation was carried out on all steps (Fig. 3). Carbon materials were obtained from commercial sources. It is important to note, that to observe the effect of structure, one needs a material with open, non-shielded zones of different reactivity^29^. The Pd_2_dba_3_ complex was synthesized by a previously described procedure^30^. The purity of the synthesized Pd_2_dba_3_ was confirmed by NMR spectroscopy and elemental analysis^30^.

The reaction of palladium deposition.

1 $$P{d}_{2}db{a}_{3}+C\to Pd/C+dba$$

**Fig. 4:**
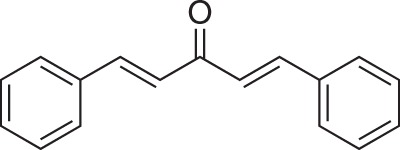
The chemical structure of dba — dibenzylideneacetone.

There are two main ways to confirm the completeness of the reaction (Equation 1): visually and by NMR. First, the solution after the deposition process looked completely transparent indicating that no deep red colored Pd_2_dba_3_ was left in the solution. Second, we recorded ^1^H NMR spectrum of the supernatant. The results clearly indicate that the palladium complex was totally consumed during the reaction and the dba ligand (Fig. 4) was released in the free form.

ICP-AES was used to verify the completeness of Pd deposition on a carbon surface. Initial Pd complex (precursor of NPs) was taken in terms of 1 wt.% of pure Pd relative to the carbon support. Results of ICP-AES measurements have shown that palladium content in prepared Pd/C sample was 0.98 wt.%, which means that 99% of the palladium was deposited on carbon. In this regard, the process is very effective in terms of palladium consumption to formation of ordered structures.

To verify that the observed particles are indeed Pd particles, we performed an EDX-spectroscopy analysis. It confirmed the presence of palladium in all samples. Two images of the sample before and after the deposition show the formation of new particles. In combination, the three experiments confirm successful and complete deposition of palladium at the carbon material surface.

The samples are stable in time. The morphological stability in time has been confirmed by imaging the samples after one day, one week, one month, one year, and five years after the preparation.

The SEM measurements give stable results, that is, relative positions of the particles do not change during the measurements and are reproduced by multiple measurements. Repeatedly recorded images of the same area are identical.

The image labeling for Fig. 2 was performed by two authors of this study independently. The labeled images were reviewed by two other authors to avoid subjectivity.

## Usage Notes

The study aims at encouraging the development of comprehensive algorithms to characterize the ordering effect and quantify the degree of structural organization. This can be done in two different ways: top-down approach (prediction-based search for high-level structural organization) and bottom-up approach (positions of all particles are recognized, and the total body of data is subject to quantification).

The dataset can be used also for the training purposes in unsupervised particle recognition tasks, semantic segmentation, clustering, and classification (e.g. of different materials).

Possible algorithms for the image processing problems are convolutional neural networks. There are examples of possible network architectures for classification, or segmentation problems (supervised — U-Net^31^, unsupervised — W-Net^32^). Autoencoders^33^ (neural networks, trained to reproduce their input) can be used to map an image into a vector space. These vectors can be used further in clustering problems.

Correlations between the structuring effects and the properties of nanomaterials (including catalytic, chemical and physical properties) represent a highly important topic. To date, however, the research is severely limited by the lack of easily available algorithms and dedicated software tools for the automated analysis of such microscopy images.

## Data Availability

The example Jupyter notebook for calculation of dataset statistics and getting specific images (e.g. images of the specific sample) are available with the dataset.

## References

[1] Weilie, Z. & Zhong L. W. *Scanning Microscopy for Nanotechnology Scanning Microscopy for Nanotechnology*: *Techniques and Applications*. (Springer New York, 2007).

[2] Reimer, L. *Scanning Electron Microscopy: Physics of Image Formation and Microanalysis* 2nd edn (Springer-Verlag Berlin Heidelberg, 1998).

[3] Singh, A. K. Experimental Methodologies for the Characterization of Nanoparticles. In *Engineered Nanoparticles* 125–170 (Elsevier, 2016).

[4] Kashin AS, Degtyareva ES, Eremin DB, Ananikov VP (2018). Exploring the performance of nanostructured reagents with organic-group-defined morphology in cross-coupling reaction. Nat. Commun..

[5] Gacutan EM (2012). Nanostructured carbon-supported Pd electrocatalysts for ethanol oxidation: synthesis and characterization. Adv. Nat. Sci. Nanosci. Nanotechnol..

[6] Kashin AS, Ananikov VP (2019). Monitoring chemical reactions in liquid media using electron microscopy. Nat. Rev. Chem..

[7] Chen J, Natte K, Wu X-F (2015). Pd/C-catalyzed carbonylative C–H activation with DMF as the CO source. Tetrahedron Lett..

[8] Felpin FX, Landais Y (2005). Practical Pd/C-Mediated Allylic Substitution in Water. J. Org. Chem..

[9] Trost BM, Kaneko T, Andersen NG, Tappertzhofen C, Fahr B (2012). Total synthesis of aeruginosin 98B. J. Am. Chem. Soc..

[10] Mubeen S, Zhang T, Yoo B, Deshusses MA, Myung NV (2007). Palladium Nanoparticles Decorated Single-Walled Carbon Nanotube Hydrogen Sensor. J. Phys. Chem. C.

[11] Jia X (2013). Synthesis of Palladium/Helical Carbon Nanofiber Hybrid Nanostructures and Their Application for Hydrogen Peroxide and Glucose Detection. ACS Appl. Mater. Interfaces.

[12] Liu, X. & Astruc, D. Development of the Applications of Palladium on Charcoal in Organic Synthesis. *Advanced Synthesis and Catalysis*, 10.1002/adsc.201800343 (2018).

[13] Wijngaarden, R. J., Kronberg, A. & Westerterp, K. R. *Industrial Catalysis*. 10.1002/9783527611966 (Wiley, 1998).

[14] Grzelczak M, Vermant J, Furst EM, Liz-Marzán LM (2010). Directed Self-Assembly of Nanoparticles. ACS Nano.

[15] Juarez MF, Fuentes S, Soldano GJ, Avalle L, Santos E (2014). Spontaneous formation of metallic nanostructures on highly oriented pyrolytic graphite (HOPG): an ab initio and experimental study. Faraday Discuss..

[16] Yang RT, Wong C (1981). Mechanism of Single-Layer Graphite Oxidation: Evaluation by Electron Microscopy. Science..

[17] Evans EL, Griffiths RJM, Thomas JM (1971). Kinetics of Single-Layer Graphite Oxidation: Evaluation by Electron Microscopy. Science..

[18] Taing J, Cheng MH, Hemminger JC (2011). Photodeposition of Ag or Pt onto TiO_2_ Nanoparticles Decorated on Step Edges of HOPG. ACS Nano.

[19] Snell KE (2014). Nanoparticle Organization through Photoinduced Bulk Mass Transfer. Langmuir.

[20] Ananikov, V. P. Organic–Inorganic Hybrid Nanomaterials. *Nanomaterials*, **9**, 1197, 10.3390/nano9091197 (2019). 10.3390/nano9091197 PMC6780615 31454924

[21] Pentsak EO (2015). Spatial imaging of carbon reactivity centers in Pd/C catalytic systems. Chem. Sci..

[22] Banhart F, Kotakoski J, Krasheninnikov AV (2011). Structural Defects in Graphene. ACS Nano.

[23] Goldstein, J. I. *et al*. Scanning Electron Microscopy and X-ray Microanalysis. 10.1007/978-1-4615-0215-9 (Springer US, 2003).

[24] Seiler H (1983). Secondary electron emission in the scanning electron microscope. J. Appl. Phys..

[25] Kashin AS, Ananikov VP (2011). A SEM study of nanosized metal films and metal nanoparticles obtained by magnetron sputtering. Russ. Chem. Bull..

[26] Pentsak EO, Cherepanova VA, Ananikov VP (2017). Dynamic Behavior of Metal Nanoparticles in Pd/C and Pt/C Catalytic Systems under Microwave and Conventional Heating. ACS Appl. Mater. Interfaces.

[27] Boiko DA, Pentsak EO, Cherepanova VA, Ananikov VP (2020). figshare.

[28] Boiko DA, Pentsak EO, Cherepanova VA, Ananikov VP (2020). figshare.

[29] Sedykh AE, Gordeev EG, Pentsak EO, Ananikov VP (2016). Shielding the chemical reactivity using graphene layers for controlling the surface properties of carbon materials. Phys. Chem. Chem. Phys..

[30] Zalesskiy SS, Ananikov VP (2012). Pd_2_(dba)_3_ as a Precursor of Soluble Metal Complexes and Nanoparticles: Determination of Palladium Active Species for Catalysis and Synthesis. Organometallics.

[31] Ronneberger, O., Fischer, P. & Brox, T. U-net: Convolutional networks for biomedical image segmentation. in *Lecture Notes in Computer Science (including subseries Lecture Notes in Artificial Intelligence and Lecture Notes in Bioinformatics)*, 10.1007/978-3-319-24574-4_28 (2015).

[32] Xia, X. & Kulis, B. W-Net: A Deep Model for Fully Unsupervised Image Segmentation. Preprint at, https://arXiv.org/abs/1711.08506 (2017).

[33] Goodfellow, I., Bengio, Y. & Courville, A. *Deep Learning*. (MIT Press, 2016).

